# Role of new Immunophenotypic Markers on Prognostic and Overall Survival of Acute Myeloid Leukemia: a Systematic Review and Meta-Analysis

**DOI:** 10.1038/s41598-017-00816-2

**Published:** 2017-06-23

**Authors:** A. F. O. Costa, D. L. Menezes, L. H. S. Pinheiro, A. F. Sandes, M. A. P. Nunes, D. P. Lyra Junior, D. M. Schimieguel

**Affiliations:** 1 Department of Pharmacy, Laboratory of Hematology, Federal University of Sergipe, Aracaju, Sergipe Brazil; 2 Fleury Group, Hematology Division, São Paulo, São Paulo Brazil; 3 Department of Medicine, Federal University of Sergipe, Aracaju, Sergipe Brazil

## Abstract

Despite technological advances, the prognosis and survival of acute myeloid leukemia (AML) adult patients remain low, compared with other hematologic malignancies. Some antigens detected by immunophenotyping may soon play a significant role in the pathophysiologic, prognostic, and overall survival (OS) rate of AML patients. Therefore, we conducted a systematic review and meta-analysis of PubMed, Scopus, Science Direct, Web of Science, and the Cochrane Library (using PRISMA guidelines). We analyzed 11 studies and 13 antigens, detected through the immunophenotyping of 639 patients. From them, twelve exhibited a negative impact with AML prognosis. The meta-analysis demonstrated a high expression of AML markers, which have been associated with a decrease in survival over 10 months (RR 2.55; IC 95%; 1.49–4.37) and over 20 months (RR 2.46; IC 95%; 1.75–3.45). Knowing that the expression of immunophenotypic markers, which are not used on a routine basis, might be able to influence disease behavior, looks promising. However, they have been associated with a poor prognosis as well as a decrease in survival. This may allow for different chemotherapeutical protocols, including future studies for new therapeutic targets.

## Introduction

Acute myeloid leukemia (AML) is an aggressive hematological malignancy characterized by clonal proliferation of immature myeloid cells at various stages of maturation^1, 2^. The genetic heterogeneity of this group of hematological malignancies makes it impractical to perform initial analyses that can encompass different genes involved in AML. This makes diagnoses difficult, which can negatively influence therapeutic strategy^3, 4^. Even with major improvements in the understanding and treatment of AML over the past several years, few advances have been made in the outcomes and survival of AML patients^5, 6^. Complete remission is expected for approximately 60‒70% of adults with AML after the induction phase of treatment, but only about 25% survive three or more years with the possibility of being cured^7^. The 5-year relative survival rate for patients from birth to 19 years has been reported to be 62.8%, but declines to 5.4%, for patients older than 65 years^8^.

Multiparameter flow cytometry (MFC) immunophenotyping provides relevant information for AML diagnosis, classification, and monitoring. MFC allows identification, quantification, and lineage assessment of abnormal blast cells and disease classification according to the maturation stage to be made^9, 10^. AML presents highly heterogeneous immunophenotypic profiles, which is probably due to genetic diversity. Leukemia-associated phenotypic markers (LAIPS) are useful to discriminate between normal/reactive immature myeloid precursors from leukemic cells and is commonly used in minimal residual disease (MRD) studies. In addition, LAIPS are also associated with molecular alterations with well-recognized prognostic implications (such as CD19 expression in AML with RUNX1-RUNX1T1)^11, 12^.

Previous studies show that a great number of distinct antigens affect AML prognosis and prediction. Nevertheless, difficulties are still found in performing accurate risk stratification for diagnoses based on immunophenotypic features^13^. Improving the accuracy of prognostic assessment of AML may allow the treatment to be more specific and risk-adapted, increase the probability of cure, and minimize treatment-related morbidity and mortality^14^.

Since there are no clear recommendations and no consistent approaches to the use of new markers in the immunophenotypic panels for AML evaluations as well as for the markers’ influence on determining prognosis and survival in clinical practice, a systemic review was done. In this review, we aimed to identify relevant publications about the influence of these new monoclonal antibodies used as immunophenotypic markers in the prognosis and survival of AML patients.

## Material and Methods

A systematic review was performed based on a scientific research protocol describing the aims and methods used. Within the limitations of the research in this area, this synthesis was performed according to the Preferred Reporting Items for Systematic Reviews and Meta-Analyses (PRISMA) statement^15^.

The question of this systematic literature review was: “*Are there new immunophenotypic markers that may aid in the prognosis and survival of acute myeloid leukemia*”?

### Search Strategy

The literature search was conducted using Pubmed, Science Direct, Web of Science, Scopus and Cochrane Library databases for articles published from 2012 to 2015. In addition, the reference lists of relevant papers were searched for additional AML studies.

The following search terms consisted of a range of pertinent terms: 1) antigens CD (MeSH); 2) antigens, differentiation (MeSH); 3) biological Markers (MeSH); 4) tumor markers, 5) biological (MeSH) and prognosis (MeSH); 6) survival rate (MeSH); 7) survival analysis (MeSH) and leukemia myeloid, acute (MeSH); or 8) acute leukemia.

### Study selection

The articles found in the search were compared with the previously defined inclusion criteria to determine the relevance of the study: (1) papers published from 2012 to 2015; (2) articles published in English, Spanish, and Portuguese; (3) articles that used immunophenotyping in their methodologies; (4) articles assessing monoclonal antibodies not included in the consensus diagnostic panels for AML (Euroflow consortium^16^); (5) articles assessing AML cases; and (6) articles with available abstract and full text.

Systematic and literature reviews, meta-analysis, editorials, conference proceedings and books were excluded from the study.

Two reviewers independently evaluated the titles and abstracts of the identified publications by applying the inclusion criteria. Potentially relevant articles were retrieved in full. The final inclusion of articles into our systematic review was based on agreement between both reviewers. In case of any disagreements between the two reviewers, a third reviewer inspected the full text article and finalized the decision whether or not to use the article.

### Rating quality of individual studies

The methodological quality of each individual study was evaluated using the Strengthening the Reporting of Observational Studies in Epidemiology (STROBE) assessment scale, which consisted of 22 items^17^. High scores meant that there was sufficient information and good design. STROBE was a highly feasible and applicable method to use for evaluating systematic reviews of observational studies.

### Data extraction and management

From the included studies, information regarding several parameters was obtained: (1) journal of publication; (2) The Journal Citation Reports (JCR) impact factor; (3) location; (4) study design; (5) aim of the study; (6) number of samples analyzed; (7) AML classification; (8) most incident subtype; (9) immunophenotypic marker; (10) prognostic value; (11) first induction treatment protocol (12) follow up; (13) survival; (14) limitations; and (15) STROBE scores. For inaccessible or incomplete full texts, authors were contacted for additional information.

### Statistical analysis

A meta-analysis of the relative risks related to the probability of survival at 10 to 20 months was performed. For analysis purposes, low expression of the immunophenotypic marker was considered as absent, and high expression was considered as present.

Survival analysis or time for the event were analyzed by dichotomous data using the knowledge of the situation of all patients in the study at 10 and 20 months^18^. A contingency table was constructed (Table 1) to analyze every connection and then the relative risks were calculated using this equation:

$$\frac{Risk\,of\,the\,event\,om\,the\,group\,with\,negative\,expression}{Risk\,of\,the\,event\,om\,the\,group\,with\,positive\,expression}=\frac{a/a+b}{c/c+d}$$

**Table 1:** Contingency table (2 × 2).

	Outcome		Total
	Alive	Dead	
Negative Marker	a	b	a + b
Positive Maker	b	d	c + d
	a + c	b + d	a + b + c + d

Correlation between the presence or absence of immunophenotypic marker and the survival outcome or not.

A correction value of 0.5 was used in order to enable the statistical analysis using the absence of death in the absence of one of the immunophenotypic markers in a 10 month follow up of survival and no patient survival in the presence of the markers in a 20 month follow up of survival as effect measures^5, 19^.

The heterogeneity of the meta-analysis was assessed using the Cochran Q and Higgins I² tests. We used the relative risk as an effect measure after taking into consideration the number of people who would be alive in the absence and presence of the immunophenotypic marker expression at 10 and 20 months. Meta-effect estimates were reported and relative risks summarized with their 95% confidence intervals. The funnel graphs and regression testing of asymmetry were used to assess potential publication bias related to survival at both 10 and 20 months. The bias was considered significant at p = 0.05. All analyses were performed with the program R version 3.3.1^20^ and the “Metafor” package^21^.

## Results

### The literature search

**Figure 1:**
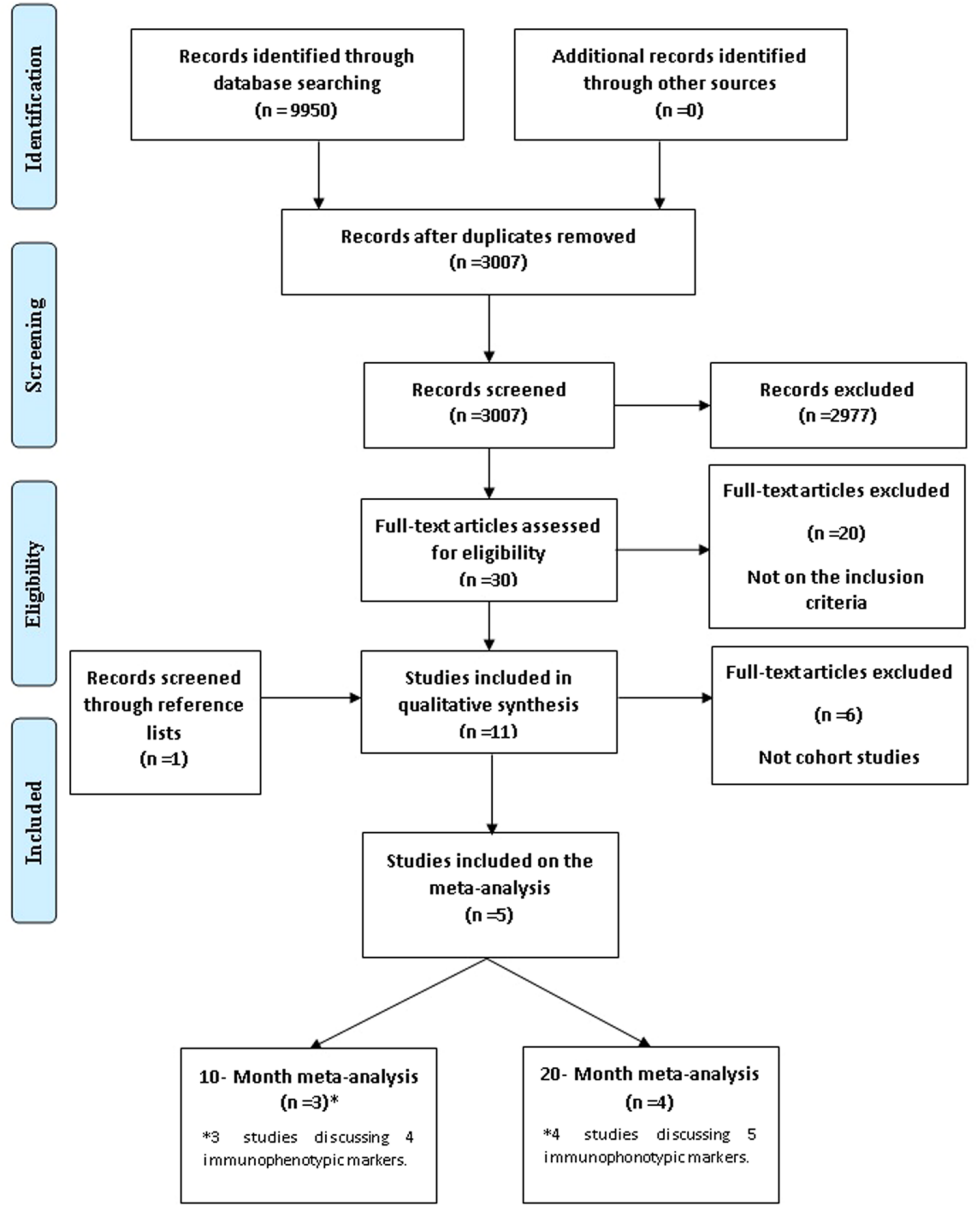
Flow diagram for study identification. Flow chart of how the research was systematically conducted for study identification.

The literature search retrieved 9,950 articles. After screening titles and reviewing abstracts, we identified 30 potentially relevant articles that focused on immunophenotypic markers and MFC (Fig. 1). In the final analysis, a total of 11 studies were included in the qualitative synthesis of this review. One of them was found after a hand search of reference lists^5, 19, 22–30^. Three of them were used on the meta-analysis for 10-month survival^19, 22, 30^ and four were used on the 20-month survival analysis^5, 19, 29, 30^.

### Study Characteristics

An overview of the characteristics of the 11 studies included in the final analysis is summarized in Tables 2 and 3. The sample size ranged from 12–142 patients with a total of 639 hematological samples analyzed in all studies^23, 24, 26, 29^. Two studies used the World Health Organization (WHO) classification for AML, six used the French-American-British (FAB) classification, and three used both FAB and WHO to classify the AML subtypes^5, 19, 22–30^. The most reported subtype in the articles was FAB M2 followed by FAB M4.

**Table 2:** Main characteristics of the individual studies analyzed on the systematic review and meta-analysis.

Marker	Article	JCR	Location	Design	Aim of the Study	STROBE
CD82	Nishioka *et al*. (Int J Cancer 2013)	5.085	Japan	Cross-sectional observational	Analyze the protein expression profile of CD34+/CD38− AML cells and compare it with the expression profile of their CD34+/CD38+ counterparts using isobaric tags for relative and absolute quantitation (iTRAQ) and explored the function of CD82 in CD34+/CD3− AML cells *in vitro* as well as *in vivo*.	17 (77.8%)
CD82	Nishioka *et al*. (Int J Cancer 2014)	5.085	Japan	Cross-sectional observational	Explore the regulation of STAT5/IL-10 by CD82 and its impact on the survival of CD34+/CD38− AML cells.	17 (77.8%)
CD87	Atfy *et al*. (Med Oncol 2012)	2.634	Germany	Cohort	Assess the prognostic significance of pretreatment detection of CD87 and the prevalence of its expression and value as a predictor for survival.	20 (91%)
CD93	Iwasaki *et al*. (Cell Stem Cell 2015)	22.268	USA	Cross-sectional observational	Report that the cell surface lectin CD93 is a functional marker of LSCs in a specific genetic subtype of AML with rearrangements of the MLL gene.	20 (91%)
CD135	Sharawat *et al*. (Cytometry B 2013)	2.398	India	Cohort	Evaluate clinical significance of FLT3 (CD135) and c-KIT (CD117) coexpression on myeloblasts in AML.	21 (95.4%)
CXCR4	Mannelli *et al*. (Cytometry B 2014)	2.398	Italy	Cohort	Investigate the expression of connexins in primary human AML cells derived from unselected patients	21 (95.4%)
CD133	Tolba *et al*. (Med Oncol 2013)	2.634	USA	Cohort	Assess CD133 expression in patients with acute myeloid or lymphoblastic leukemia and to evaluate its correlation with the different clinical and laboratory data as well as its relation to disease outcome.	20 (91%)
TRAILR2 (CD262)	Schmohl *et al*. (Anticancer research 2015)	1.826	Germany	Cohort	Evaluate the association of co-expression of TRAILR1-3, TNFR1 and FAS on AML blasts at first diagnosis with different AML subtypes and risk groups and to combine with clinical data in order to evaluate their prognostic and clinical significance.	21 (95.4%)
TRAILR3 (CD263)						
TNFR1						
ILT3	Dobrowolska *et al*. (Cytometry B 2013)	2.398	USA	Cross-sectional observational	Investigated ILT3 expression by normal and leukemic myeloid precursors. We report that ILT3 expression identifies normal hematopoietic precursors committed to the monocytic lineage and that ILT3 is a reliable marker that distinguishes AML with monocytic differentiation from other types of AML	19 (86.4%)
hMICL	Larsen *et al*. (Cytometry B 2012)	2.398	United Kingdom	Case control	Based on data from stem cell research, they hypothesized that the human inhibitory C-type lectin like receptor (hMICL) might represent a novel diagnostic and prognostic vehicle in a standard flow cytometry (FCM) setting.	20 (91%)
CD90	Chávez-gonzález *et al*. (Arc Med Res 2014)	2.645	Mexico	Case control	Analyze the expression of CD90, CD96, CD117, and CD123 on CD34+ CD38− cells, CD34+ CD38+ cells and CD34- CD38+ cells.	21 (95, 4%)
CD96						

AML: Acute myeloid leukemia; JCR: journal citation reports; iTRAQ: isobaric tags for relative and absolute quantification; STAT5: signal transducer and activator of transcription 5; IL-5: interleukin 5; LSCs: leukemic stem cells; MLL gene: mixed lineage leukemia gene; FLT3: fms related tyrosine kinase 3; c-KIT: receptor tyrosine kinase protein; TRAILR1-3: Tumor necrosis factor-related apoptosis-inducing ligand 1-3; TNFR1: Tumor necrosis factor receptor 1; FAS: cell surface death receptor; ILT3: immunoglobulin-like transcript 3; HSC: hematopoietic stem cell.

**Table 3:** Main disease and treatment features of the individual studies included on the systematic review and meta-analysis.

Marker	Patients (n)	Classification	Treatment	Prognosis	Follow-Up	Cut-Off	Survival	Gene mutation
CD82	12	AML with myelodysplasia changes: 4	NR	POOR.	NR	NR	NR	NR
CD82	14	M4: 4MDS transformed to AML: 4	NR	POOR	NR	NR	NR	NR
CD87	110	M4: 36	Double-induction therapy with thioguanine, cytosine arabinoside (AraC), and daunorubicin (TAD) followed by high-dose Ara-C and mitoxantrone (HAM). M3 cases received therapy protocols that contained all-trans-retinoic-acid (ATRA).	POOR	17 months	>25% of leukemic cells	High expression of CD87 predict shorter overall survival.	NR
CD93	36	Normal: 11	NR	POOR	NR	NR	NR	NR
CD135	115	M2: 66	“3 + 7 “(Daunorubicin and cytosine arabinoside) with Daunorubicin at 60 mg/m2 for 3 days.	POOR	15.5 months	>20% of myeloblasts	High expression of CD135 predicted poor EFS and OS	FLT3 ITD – 17%
CXCR4	142	M2: 38 M4: 38	“3 + 7” (Cytarabine 100 mg sqm21 over 3-h intravenous infusion bid on days 1–7; Idarubicin 12 mg sqm21 30 min intravenous infusion on days 1–3).	POOR	20 months	>13, 16 MFI	High expression of CXCR4 predict shorter overall survival.	FLT3 ITD – 23, 9% NPM1 MUTATED – 39, 4% CEBPA MUTATED – 11, 3%
CD133	30	NR	“3 + 7” induction chemotherapy protocol: doxorubicin (30 mg/m2/day) for 3 days and cytarabine (100 mg/m2/day as a continuous 24-h intravenous infusion) for 7 days.	POOR	12 months.	>10% of blast cells	Increased CD133 leads to decrease the survival by the time.	NR
TRAILR2 (CD262)	46	M2: 17	Anthracycline-based induction therapy (Idarubicin or daunarubicin) and other approved or supportive therapies.	POOR	55–120 months	>3, 2 SFI	Cut-off analyses for TRAILR2 showed significantly shorter overall survival	NR
TRAILR3 (CD263)				GOOD		NR	Cut-off analyses for TRAILR3 showed a increase in survival.	NR
TNFR1				POOR		>3, 2 SFI	Cut-off analyses for TNFR1 showed significantly shorter overall survival	NR
ILT3	37	M4/M5: 18	NR	POOR	NR	>10% of leukemic population	NR	NR
hMICL	55	M4: 7 M2: 7 ND: 29	NR	POOR	NR	>25% of CD45low/SSclow	NR	NR
CD90	12	M2: 4	First course of induction: ATEDox 5) cytarabine, mercaptopurine, doxorubicin) and second identical induction course.	Undetermided	4-45moths	NR	NR	NR
CD96				POOR		NR	NR	NR

AML: Acute myeloid leukemia; TRAILR2: Tumor necrosis factor-related apoptosis-inducing ligand 2; TRAILR3: Tumor necrosis factor-related apoptosis-inducing ligand 3; TNFR1: Tumor necrosis factor receptor 1; hMICL: human myeloid inhibitory C-type lectin-like receptor; MDS: myelodysplastic syndrome; MFI: Mean Fluorescence intensity; SFI: Specific fluorescence indices; NR: not related; ATEDox: cytarabine, 6-thioguanine, etoposide, doxorubicin; EFS: Event-Free Survival; OS: Overall survival.

Most studies were performed in countries with high scientific and technologic development, including the United States (US), Germany, and Japan^5, 19, 22–25, 27^. The Journal Citation Reports (JCR) impact factor found in the search ranged from 1.826 to 22.268^19, 25^. There were only two pediatric studies, and the others focused on adults^26, 30^. The expression of 13 different antigens was evaluated in all articles (Table 4). All 11 studies were based on proving the impact of novel antigens on AML prognosis while five of them were cohort studies that also proved the influence of these markers on patient survival^5, 19, 22, 29, 30^. The most common chemotherapy regimen was anthracycline-based induction therapy (3 + 7), although other alternative treatments were chosen by some groups.

**Table 4:** Basic features of each antigen analyzed on this systematic review and meta-analysis.

Antigen	Molecular Group	Function	Frequency in AML	Association with specific disease features	Prognostic Impact
**CD82**	**A member of tetraspanin superfamily**	**Cell adhesion**	**NR**	**NR**	**Poor**
CD87	Urokinase plasminogen activator receptor	Conversion of plasminogen to plasmin	72.2%	FAB M4 and M5	Poor
**CD93**	**C-type lectin transmembrane receptor**	**Phagocytosis, inflammation, and cell adhesion**	**NR**	**Leukemia Stem Cells in AML with rearrangements of the MLL gene**	**Poor**
CD135	Tyrosine kinase receptor	Promote the growth and differentiation of primitive hematopoietic cells	82%	NR	Poor
**CXCR4**	**Receptor for stromal-derived factor 1 (SDF1)**	**Cell adhesion and hematopoietic stem cell niche regulation**	**50%**	**Hepato-splenomegaly and extra-hematological disease**	**Poor**
CD133	A novel five transmembrane molecule	Regeneration, proliferation and differentiation of Steam cells	56%	FAB M4 and M5	Poor
**CD262**	**Death receptor**	**Apoptosis**	**21, 7%**	**Monocytic subtipes, AML FAB M5 and M6**	**Poor**
CD263	Death receptor	Inhibition of cell death through competitive binding activity	NR	AML FAB M0	Good
**CD120a**	**Death receptor**	**Mediation of cytotoxicity; signaling of fibroblast growth, endothelial activation/adhesion**	**21, 7%**	**AML FAB M2 and M5**	**Poor**
ILT3	Immunoglobulin-like transcript (ILT) 3	Inhibitory receptor: down-regulation of immune responses.	83%	AML with monocytic differentiation and microgranular acute promyelocitic leukemia	Poor
**hMICL**	**A glycosylated transmembranal C-type lectin**	**Control of myeloid cell activation during inflammation**	**89%**	**Poorly characterized CD34 negative patient group**	**Poor**
CD90	Cell-surface glycoprotein	Proliferation and expansion processes	NR	NR	Undetermined
**CD96**	**Member of the immunoglobulin superfamily**	**Adhesive interactions of activated T and NK cells during the late phase of the immune response**	**NR**	**NR**	**Poor**

FAB: French-American-British Classification; AML: Acute myeloid leukemia; TRAILR2: Tumor necrosis factor-related apoptosis-inducing ligand 2; TRAILR3: Tumor necrosis factor-related apoptosis-inducing ligand 3; TNFR1: Tumor necrosis factor receptor 1; hMICL: human myeloid inhibitory C-type lectin-like receptor; NK: Natural Killer; NR: not related.

The methodological quality of observational studies conducted with the STROBE tool showed that eight articles scored >90% revealing a high methodological quality of the included studies. One article scored 19 points, which was equivalent to 86.4%, four scored 20 points, which was equivalent to 91%, and four scored 21 points, which was equivalent to 95.4%^5, 19, 22, 25–30^.

### Prognostic value and survival

Most articles showed that the expression of the evaluated antigens has a negative impact on prognosis of AML. Only one article showed that CD263 has a positive value on prognosis, and two articles showed that the values of CD90 and ILT3 were not determined^19, 26, 27^. All five cohort articles showed a decreased survival related to the high expression of the immunophenotypic markers.

Two articles by the same authors discussed CD82, a member of the tetraspanin superfamily that was originally identified as an accessory molecule in T-cell activation and in nonimmune cells in integrin-mediated cell adhesion to the extracellular matrix. Both articles evaluated the expression of CD82 in the self-renewing leukemia stem cell (LSC) compartments (CD34^+^/CD38^−^ cells) and the CD34^+^/CD38^+^ compartments of AML cells. They showed that LSC expressed a higher amount of CD82 than CD34+/CD38+ AML cells; these findings suggested that overexpression of CD82 may render LSC able to adhere to the bone marrow (BM) niche where it appears to regulate maintenance of leukemia stem cells within the BM niche. In addition, they showed that down-regulation of CD82 in LSC may stimulate mobilization of these cells from the BM niche to PB and sensitize them to chemotherapeutic agents^23, 24^.

CD87 is a urokinase plasminogen activator receptor that initiates the conversion of plasminogen to the protease plasmin. CD87 is involved in signal transduction of cytoplasmic signals to the cytoskeleton. Atfy *et al*., showed that high expression of this antigen is associated with a decrease in AML patients’ overall survival. Regarding prognostic values, the authors presented an association of CD87+ with clinical features; this association predicted a more aggressive course of the disease with a negative prognostic impact. They suggested that CD87 expression should be included in the initial diagnostic AML work-up^5^.

CD93 is a C-type lectin transmembrane receptor that is involved in the modulation of phagocytosis, inflammation, and cell adhesion. Using flow cytometry, Iwasaki *et al*. evaluated the CD93 expression profile on CD34^+^CD38^−^ cells of various AML subtypes and normal cord blood. They observed that CD93 was expressed on a significant percentage of cells in the LSC fraction of MLL-rearranged leukemia, while this marker was negative on LSC subpopulations within non-MLL leukemia and normal cord blood cells. Since MLL rearrangement is associated with a dismal prognosis in acute leukemia, expression of CD93 in the LSC compartment of AML cases may be a useful surrogate marker to identify this AML subgroup^25^.

CD135 (FLT3) is a tyrosine kinase receptor that has a significant role in leukemogenesis. Sharawat *et al*. evaluated CD135 and CD117 expressions in a cohort of 115 AML patients (64 pediatric and 51 adults) and showed that CD135 was expressed in 82% of all cases. There was no association of CD135 expression with the FLT3 internal tandem duplication mutation, a molecular abnormality associated with unfavorable AML prognosis. Nevertheless, co-expression of CD135 and CD177 was associated with a decrease in event free survival (EFS) and overall survival (OS) in multivariable analysis in both age groups^30^.

Mannelli *et al*. investigated the expression of CXCR4 (CD184) in AML. CXCR4 is a receptor for stromal-derived factor 1 (SDF1) that plays a very important role in hematopoiesis development and organization of immune system. After analyzing whole blasts and CD34^+^ cells in 142 adult non-M3 AML cases, the authors used mean fluorescence intensity (MFI) and showed a correlation between high CXCR4 expression and decrease in EFS and OS in addition to an unfavorable prognosis. In addition, CXCR4 expression was associated with high leukemic burden, as estimated by DHL level and white blood cell and peripheral blast cell counts^29^.

CD133 is a novel five transmembrane molecule expressed on primitive normal hematopoietic progenitors. Tolba *et al*. evaluated the expression of this antigen in 30 AML cases and 30 acute lymphoblastic leukemia patients and observed that CD133 was expressed in 56.6% of AML (n = 17). The authors observed an important correlation between the expression of CD133 and the survival of AML patients and demonstrated a decrease in OS with an increase in CD133 expression. As for the prognostic value, it was concluded that CD133 expression was highly associated with poor prognosis in AML patients^22^.

In 46 AML patients, Schmohl *et al*. assessed the expression of the death receptors, including TRAILR1, 2, and 3 (CD261, 262, and 263, respectively), TNFR1 (CD120a), and FAS (CD95). CD262 is involved in induction of apoptosis in lymphoid cells. CD263 is a marker of neutrophilic granulocytes that participates in apoptosis regulation and inhibition of cell death through competitive binding activity. TNFR1 is expressed on monocytes, lymphocytes, and granulocytes, and is involved in cytotoxicity mediation. The authors concluded that CD262 and TNFR1 expressions showed significantly shorter OS, earlier disease onset, and higher probability of relapse in AML patients. Conversely, CD263 expression exhibited an enhanced OS. As for prognostic value, high expressions of CD262 and TNFR1 were found to be associated with unfavorable prognostic groups, and CD263 was found in cases with favorable risk^19^.

Dobrowolska *et al*. evaluated one member of the immunoglobulin-like transcripts (ILT3 expression) in normal and leukemic myeloid precursors in 20 healthy individuals and 37 AML cases. ILT3 is a member of the large family of ILT molecules, leukocyte Ig-like receptors (LIRs), and monocyte/macrophage Ig-like receptors (MIRs). The authors showed that ILT3 was expressed in all cases of AML displaying monocytic differentiation but not in the AML subtypes M1/M2 and M3. It was shown that expression of ILT3 by leukemic cells contribute to the inhibition of tumor specific T cell responses. As for the prognostic impact, frequent cytogenetic abnormalities observed in AML patients with ILT3+ were those associated with intermediate prognosis, but it’s potential value as a prognostic marker, particularly in cytogenetically normal AML, remains to be determined^27^.

Larsen *et al*. evaluated hMICL expression in one article in 93 AML patients. hMICL is a heavily glycosylated transmembrane C-type lectin with an unknown function. Cryopreserved mononuclear cells and bone marrow samples were used in the analysis. They showed that hMICL was found to be restricted to the CD45low/SSClow population of AML cells. The authors suggest that no loss of hMICL expression in AML patients may be an early indicator of relapse. In addition, hMICL preserved fluorescence intensity at relapse, suggesting that this antigen could be a tool for minimal residual disease quantification by flow cytometry in AML^28^.

CD90, a cell-surface glycoprotein, seems to be involved in proliferation and expansion processes, and CD96 is a member of the immunoglobulin superfamily. In one article, Chaves-Gonzales *et al*. evaluated the expression of these two antigens in two distinct primitive cell population compartments from bone marrow. CD34+ CD38− cells (enriched for HSC) and CD34+ CD38+ cells (enriched for HPC) obtained from 12 pediatric AML patients were used for the analysis. The results showed that CD90 showed slight incremental increases in patients who reached remission and did not relapse. As for CD96, the authors describe greater expression in relapse and higher levels in AML cells than in normal bone marrow cells before and after chemotherapy^26^.

### Meta-analysis

Five cohorts were analyzed at first, in which the results of quantitation of six immunophenotypic markers were evaluated^5, 19, 22, 29, 30^. The expression of these markers are considered independent events, since their pathogenic significance are not necessarily interconnected within certain pathways. The survival analysis over 10 months showed a great heterogeneity with a significant Cochran Q test (Q [df = 5]; = 11.3330; p = 0.0452) and the Higgins I² test showed a result of 83.33%, indicating high heterogeneity and suggesting an impediment to achieving the meta-analysis. Despite that impediment, the tests were still performed showing a meta-analytical estimate of no significant risk (1.49). The funnel plot and regression testing for evaluation of publication bias showed a large asymmetry (t = 5.0896; df = 4; p = 0.007) (Supplementary Figs S1‒2).

Therefore, two studies were considered responsible for the asymmetry in the analysis and withdrawn of the analysis in 10 months, remaining four events in the three articles included^5, 19, 30^. On the 20-month analysis, one article was removed for not having information regarding 10-month survival, remaining five events in the four articles included^5, 19, 29, 30^.

The Q Cochran test for 10 months survival showed that the studies included in the meta-analysis would be homogeneous (Q (df = 3) = 1.0512; p = 0.7889), and the Higgins I² test showed a result of 0% indicating no heterogeneity. The same results were found in relation to the markers collected at 20 months, both in relation to Q Cochran test (Q (df = 4) = 2.6083; p = 0.6254) and the Higgins I² test (0%). It was decided then, to perform the two meta-analyses using the fixed effects model.

**Figure 2:**
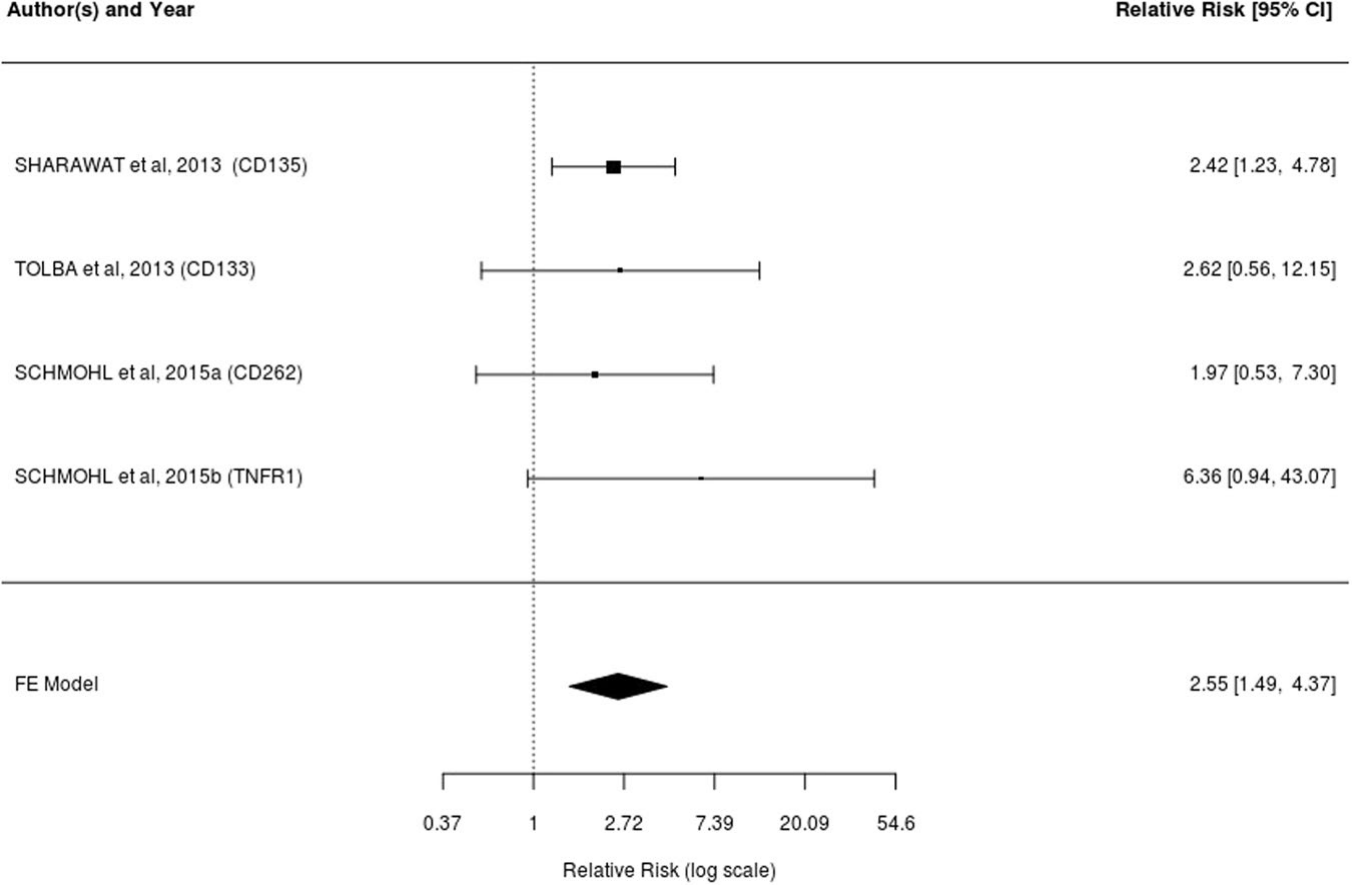
Forest Plot of relative risks and confidence intervals of 10-month survival. Relative risks and confidence intervals of survival at 10 months after the withdrawal of the two studies that caused the asymmetry, associated with the non-detection/detection of the immunophenotypic markers in each study and its meta-analytical measurements.

**Figure 3:**
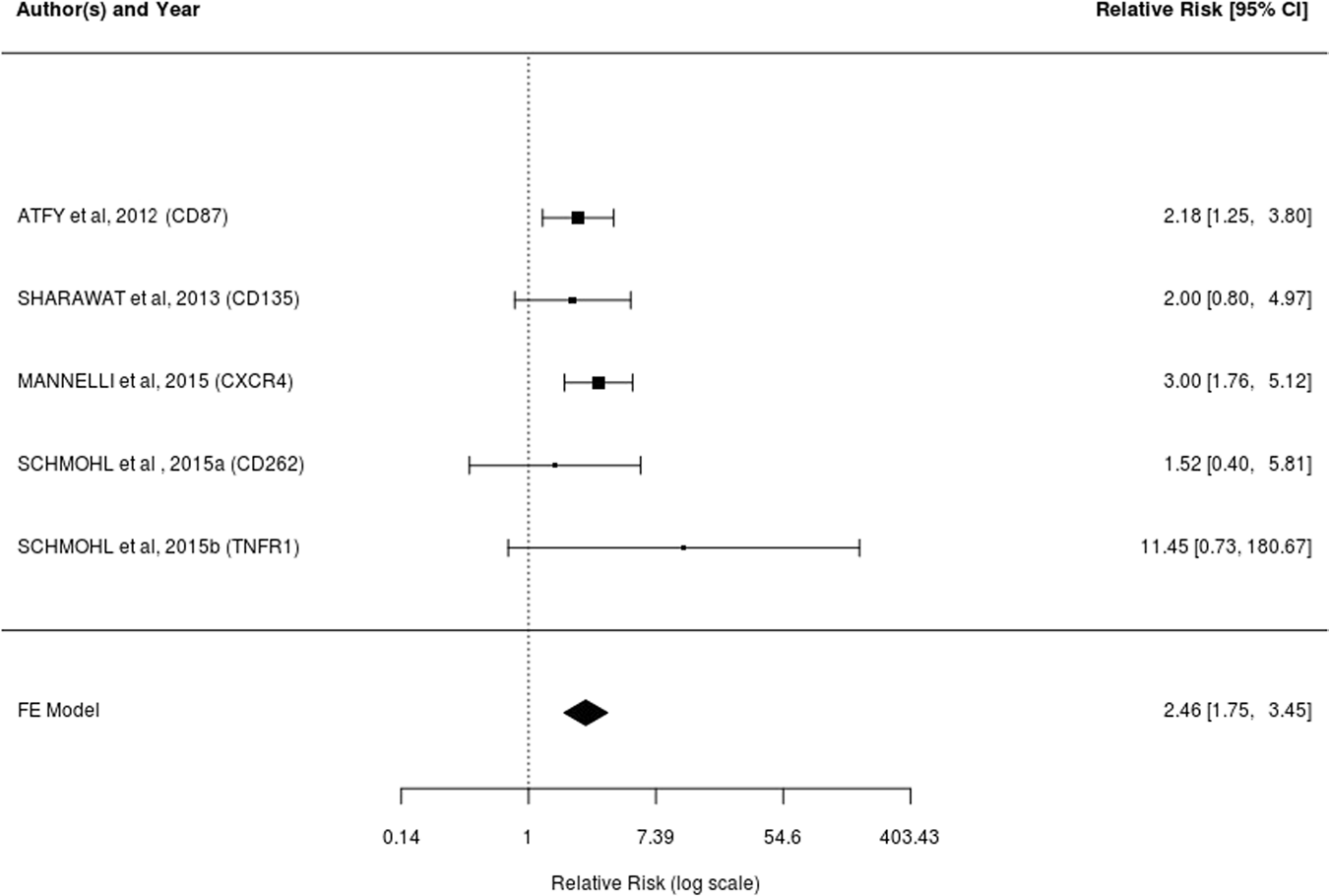
Forest Plot of relative risks and confidence intervals of 20-month survival. Relative risks and confidence intervals of survival at 20 months associated with the non-detection/detection of the immunophenotypic markers in each study and their meta-analytical measurements.

The meta-analytical estimate represented by the relative risks of survival rate at 10 months (Fig. 2) was 2.55 (95% CI: 1:49‒4:37) when analyzed for the risk of survival in the absence of the immunophenotypic marker expression and its relationship with the risk of survival in the presence the immunophenotypic marker expression. The estimate at 20 months (Fig. 3) was 2:46 (95% CI: 1.75‒3.45). Both sets of values were significant.

### Publication bias potential

After resetting the final models, especially the 10-month survival, the funnel plot and the regression test for analysis of the asymmetry were used to assess the potential for publication bias. Both tests showed that publication bias was not significantly associated with both the 10 and 20 month survival (t = 0.9055, df = 2; p = 0.4608 and t = 0.2818; df = 3; p = 0.7964, respectively) (Supplementary Figs S3–4).

## Discussion

MFC has an important role in AML diagnosis, classification, and evaluation of treatment effectiveness. Over the last several years, important achievements were obtained in the field, including improvements in flow cytometry instrumentation (>8 colors) and new analytical strategies. However, the discovery of new immunophenotypic markers for AML diagnosis was limited, and immunophenotypic panels have remained similar over the last twenty years. Therefore, there is still a need for new markers for leukemic myeloid cells to be included in clinical routines to increase the value of MFC not only as a diagnostic but also prognostic tool for monitoring of MRD and for development of drugs for targeted therapy^31^.

In this systematic review, we observed that some markers could be used for AML diagnosis and during the follow up. For example, the high expression of CD87 is an indicator of morphologically and antigenically poorly differentiated disease, especially in the M4 and M5 subtypes^5^. The inhibitory receptor ILT3 is a highly sensitive and specific marker for both diagnosis and monitoring of AML with monocytic differentiation; ILT3 as a marker is particularly useful in the differential diagnosis of AML with monocytic differentiation and microgranular acute promyelocytic leukemia, two leukemia subtypes that require different treatment strategies^27^. Another marker, hMICL, expressed significantly higher fluorescence intensity compared to normal bone marrow, suggesting suitability of this antigen as a pan-AML marker^28^. Regarding cell adhesion, CD82 has been shown to have a high expression in AML cells suggesting an increase of adhesion to the BM niche. On the other hand, down-regulation of CD82 in AML cells may stimulate circulation of these cells into peripheral blood^23^.

Many prognostic factors have been established in the past few decades in AML, including age, cytogenetic abnormalities, white blood cell count, serum lactate dehydrogenase, and the presence of antecedent hematologic disorder. This systematic review has shown that the high expression of CD87 on peripheral blood blasts was associated with relapse and poor prognosis and could be incorporated into the initial diagnostic work-up of AML patients^5^. CXCR4 could be also included due to its high expression on the surface of the entire leukemic population and demonstration of its influence on the overall CR rate and as poor prognostic factor for DFS and OS^29^. Another interesting study described the association between high expression of CD93 and loss of CDKN2B (p15Ink4b) expression; a cell-cycle inhibitor gene that has been shown to play a prominent role in leukemia pathogenesis and correlate with poor prognosis^25^.

The demonstration that the high expression of these immunophenotypic markers influence not only the survival of AML patients but also the prognosis, brings up some questions. Since immunomarkers are signaling those important prognostic data and it is known that molecular mutation can also have influence on the disease pathways regarding both survival and prognostic features, how these two information can be combined on determine better accurate prognostic factors, and what association could be found between them? We could observe that this data are rarely combined on the articles included on this systematic review and meta-analysis. It was evidenced that majority of patients that expressed high CXCR4 also presented the NPM1 mutated gene, what gives it a worst prognosis^29^. Another study showed that FLT3 receptor (CD135) was associated with the FLT3 Internal tandem duplication (ITD) mutation, which confers a poor prognosis^30^. Since gene mutations are of utmost importance in deciding the patients’ risk-stratification, the knowledge of this association can bring advancement on treatment decision and give even more precision to it, raising the chances of cure. From this preliminary information, it is possible to envision future studies involving the connection of these two types of markers to predict prognosis and survival of AML.

Despite the advancement of therapeutic progress, the overall survival of AML patients remains low^5^. One method to attempt to increase these patients’ survival could be a more specific chemotherapeutic protocol for this heterogeneous group of diseases. It is also necessary to identify in advance those patients who have greater resistance to treatment, higher relapse rates, and lower survival. We performed a meta-analysis using the fixed effects model. Four new immunophenotypic markers, CD133, CD135, TRAIL2 (CD262), and TNFR1 (CD120a) were demonstrated and showed correlation with lower survival at 10 and 20 months. Thus, these antigens can be used as early markers in combination with other prognostic factors for risk stratification of relapse. CD133 antigen appears to be expressed restrictively in the more immature cell population, and examination of the articles has made it possible to observe that expression of the CD133 in acute leukemia could be correlated with an immature phenotype of the myeloid blasts and highly associated with poor prognosis^5^. Proliferation regulators (such as tyrosine kinase receptors) play an important role in the pathogenesis of acute myeloid leukemia. The high expression of CD135 was associated with lower EFS and OS^30^. As verified by this study, the expression of death receptors is typically associated with the apoptotic regulation of leukemic blasts that demonstrated a significant association of TRAILR2 expression on blasts from patients in adverse risk groups and showed a negative impact on overall survival. Results concerning TNFR1 showed that this receptor is for the immune-modulating cytokine TNF. TNFR1 may play a role in initiation and proliferation of AML blasts due to its high expression, which appears to be related to a lower survival in AML patients^19^. Technically, this meta-analysis demonstrated that meta-analytical estimates, represented by the relative survival risk at 10 and 20 months, were both significant when the risk of survival in the absence and presence of the markers was analyzed. Notably, in our analysis, there was no heterogeneity among the cohort studies, which implied that the present method to combine the results from these studies was reasonable. Thus, the conclusions from this analysis should be credible. However, due to the relatively limited number of cases included in these studies, further analyses of larger series of patients are needed to confirm these preliminary observations.

Minimal residual disease is a term used to describe detection of subclinical levels of leukemia using multiparameter flow cytometry or molecular-based approaches. Employing the MFC for minimal residual disease detection appears to be a reliable method for obtaining rapid and objective patient remission status, provide early end points in clinical trials, and to inform patient management of a patient’s status. Emerging evidence indicates that MRD detection in patients with AML is also associated with poor prognosis, and early therapeutic interventions may be of clinical benefit. In addition, other studies have reported on the progression of new drug development that target specific areas of the leukemia cells; this is important since the current treatment can help, but does not always cure, AML patients. In this review, we observed that some antigens can be useful both as a marker for MRD and potential therapeutic target. ILT3 is an antigen expressed in AML displaying monocytic differentiation that supports differentiation and subsequent AML diagnosis of AML. It may also be a candidate marker for MRD detection in AML patients due to its high sensitivity, specificity, and stable expression. This antigen also can be a target for therapy in AML with monocytic differentiation as a result of inhibition of ILT3 signaling with specific antibodies or antagonists, which may render ILT3+ AML cells more susceptible to differentiation agents and anti-tumor T cell responses^27^. CD93, an AML with MLL rearrangements marker expressed on leukemia stem cells (LSC), may also be a useful therapeutic target candidate for anti-LSC therapy and prognostic marker for quantitation of minimal residual disease^25^. hMICL is a pan-AML marker uniformly present on cells. It can be poorly immunophenotypically lineage-characterized and difficult to monitor for residual disease and development of drugs for targeted therapy^28^. The glycoprotein CD82 could be an attractive target for LSC eradication, due to its important role in regulation of the AML cell survival and their adhesion to bone marrow microenvironment^23^.

There are some limitations that should be noted in our systematic review and meta-analysis. One of the most important is the lack of actual patient data included in the analysis. Despite that, it is known that AML is a disease with low incidence. In the US, the estimate for 2024 predicts that leukemia, including AML, will represent 3% of all cancers in male and females^8^. It was notable that some data discrepancies were found as in the case with hMICL, in which the author associated its high hMICL expression with relapse, but its role in the prognosis was not clear. More data are necessary to establish the extent that hMICL-based immunophenotyping can detect treatment failure, which is common in most AML patients. The same results were observed for CD90 and CD96, in which the author also shows an association of its high expression with relapse, but does not associate either CD90 or 96 with prognosis. CD96, especially, does not demonstrate high fluorescence in the first diagnosed sample, only in the normal bone marrow sample.

Regarding the limitations of our systematic review and meta-analysis, survival information was extracted from survival curves and not from a mortality table. Secondly, positive and negative expression were considering basing on the MFI of each study Third, the criteria to determine the positive or negative antigen expression varied across the included studies. Fourth, from the six immunophenotypic markers included in the meta-analysis, two were removed as they caused heterogeneity between the studies, which may have been due to some methodological differences. Fifth, we only searched for articles published in English, Spanish, and Portuguese and may have missed relevant publications in other languages. Finally, some studies included in the qualitative synthesis also evaluated M3 AML patients, a subgroup recognized for its excellent prognosis. However, only two studies included in the meta-analysis contained M3 AML cases, both with a low number of patients (<10%, Table 3) and did not cause any heterogeneity on the analysis.

Despite the limitations listed above, the present analysis revealed the prognostic value of new antigen expression in AML. Although the high expression of CD133, CD135, TRAIL2 and TNFR1 was not significant in most of the individual studies regarding the occurrence of the outcome, when they were collected in the meta-analysis a high correlation was observed with poor prognosis and low DFS and OS. However, further prospective studies with larger sample sizes are required to include these new immunomarkers in MFC routine of acute myeloid leukemia.

## Electronic supplementary material

Supplementary Informationpdf
