# Supplementary material for: Role of new Immunophenotypic Markers on Prognostic and Overall Survival of Acute Myeloid Leukemia: a Systematic Review and Meta-Analysis
Source: Sci Rep. 2017 Jun 23;7:4138. doi: 10.1038/s41598-017-00816-2 (PMC5482890; doi:10.1038/s41598-017-00816-2)
Supplement: Supplementary file 1 — Supplementary Informationpdf [file 41598_2017_816_MOESM1_ESM.pdf]

ROLE OF NEW IMMUNOPHENOTYPIC MARKERS ON PROGNOSTIC AND OVERALL SURVIVAL OF ACUTE MYELOID LEUKEMIA: A SYSTEMATIC REVIEW AND META-ANALYSIS

Costa AFO[1], Menezes DL[1], Pinheiro LHS[1], Sandes AF[2], Nunes MAP[3], Lyra Junior DP[1], Schimieguel DM[1]

[1]Department of Pharmacy, Laboratory of Hematology, Federal University of Sergipe, Aracaju, Sergipe, Brazil.

[2]Fleury Group, Hematology Division, São Paulo, São Paulo, Brazil.

[3]Department of Medicine, Federal University of Sergipe, Aracaju, Sergipe, Brazil.

*Corresponding author:

amandafernandes@hotmail.com (AF)

Supplementary Figure S1. Forest plot with relative risks and confidence intervals of survival in 10 months.

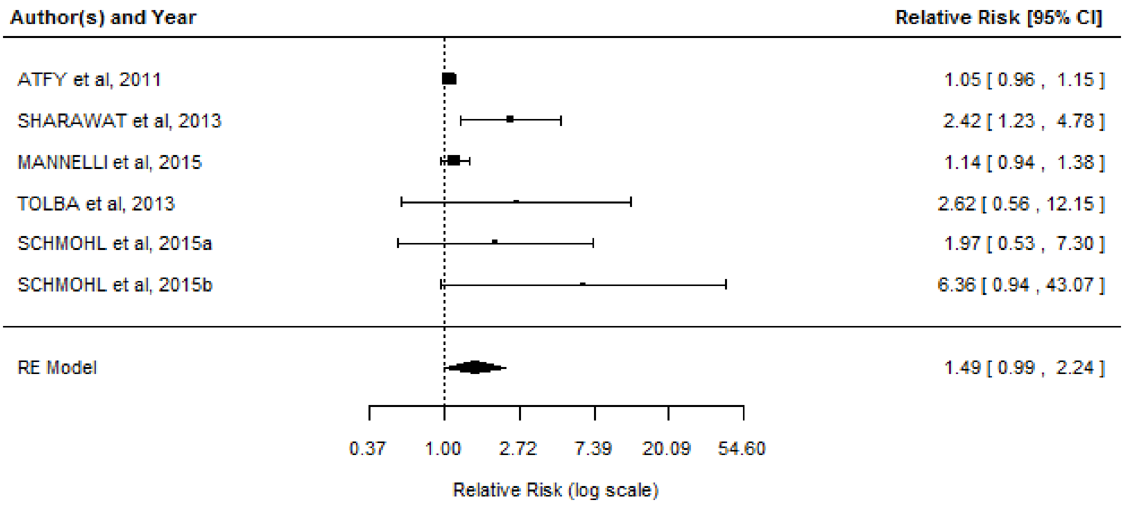

Relative risks and confidence intervals of survival at 10 months associated to the non-detection / detection of the immunophenotypic markers in each study and its meta-analytical measurements before the withdraw of the two studies found to be responsible for the asymmetry.

Supplementary Figure S2. Funnel plot of studies on survival in 10 months.

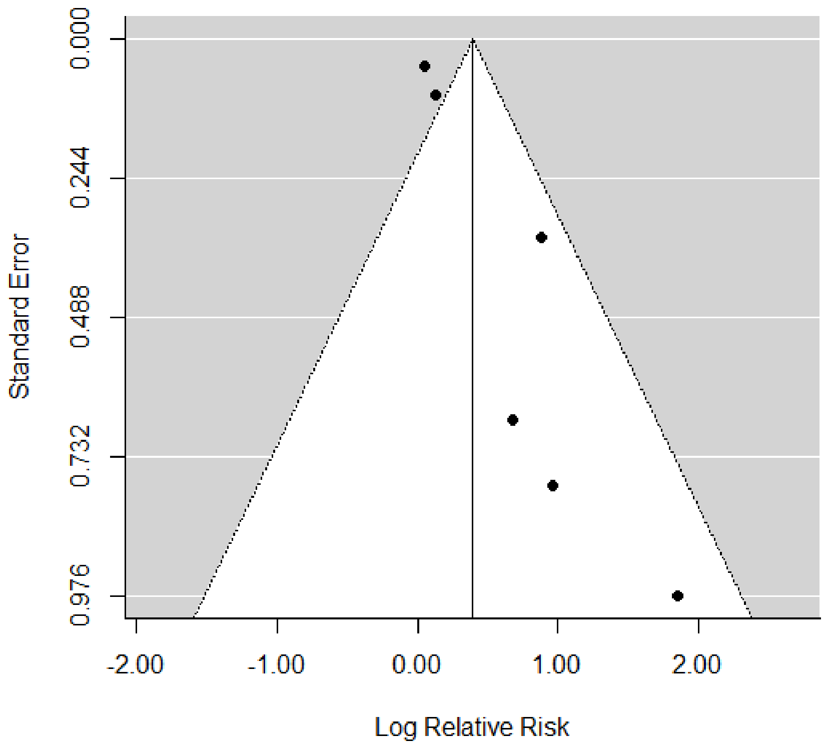

Publication bias potential of 10-month survival associated with the non-detection / detection of the immunophenotypic markers in each study and its meta-analytical measurements before the withdraw of the two studies found to be responsible for the asymmetry.

Supplementary Figure S3. Funnel plot of studies on survival in 10 months

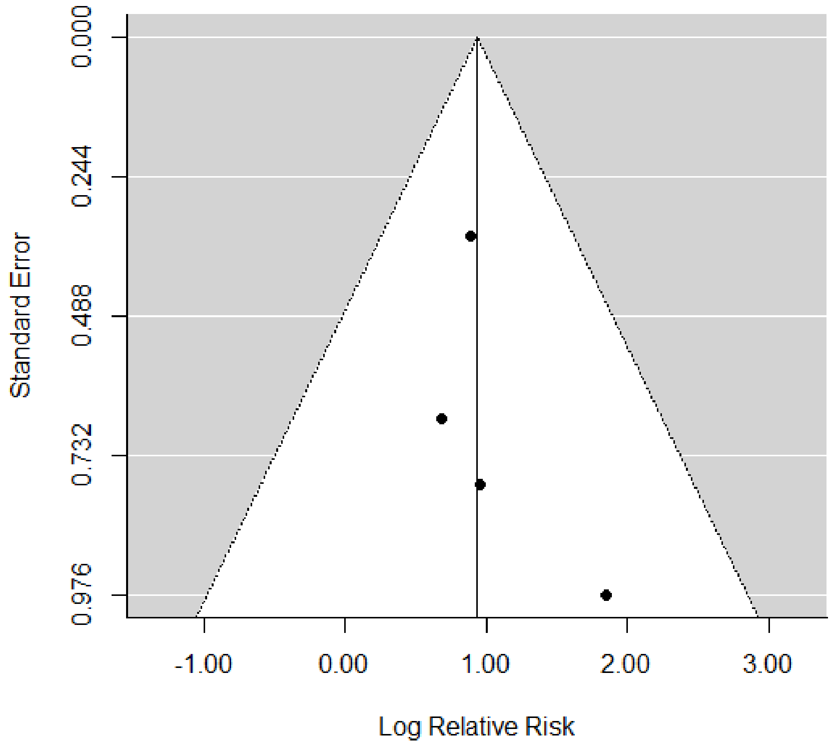

Publication bias potential of 10-month survival associated with the non-detection / detection of the immunophenotypic markers in each study and its meta-analytical measurements after the withdraw of the two studies found to be responsible for the asymmetry.

Supplementary Figure S4. Funnel plot of studies on survival in 10 months.

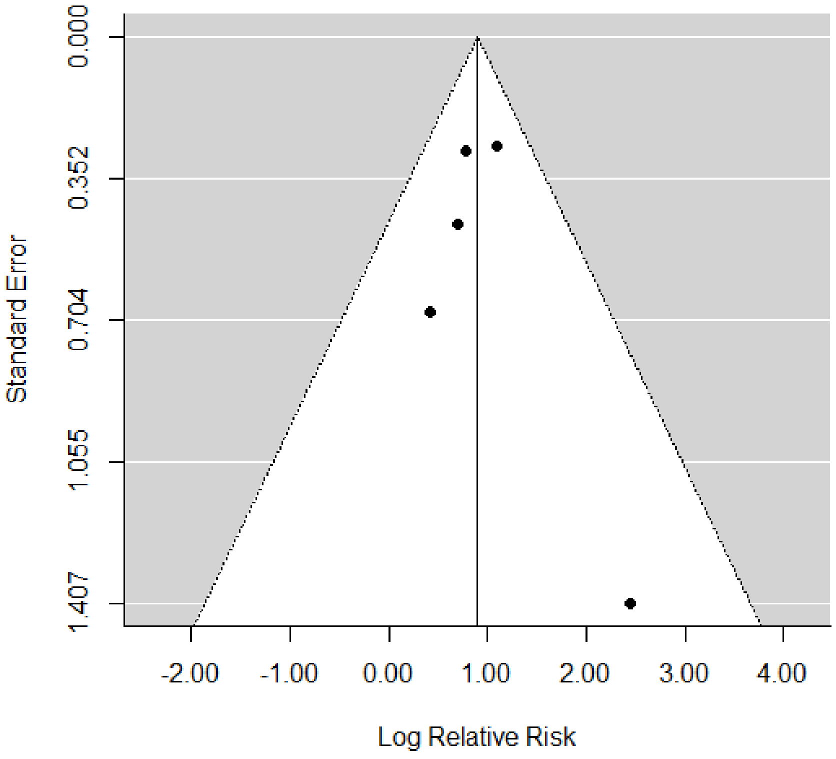

Publication bias potential of 20-month survival associated with the non-detection / detection of the immunophenotypic markers in each study and its meta-analytical measurements.
